# An Essay on the Structure and Development of the Dental Tissues: Presented to the Faculty of the Baltimore College of Dental Surgery, for the Degree of Doctor of Dental Surgery, March 1st, 1854

**Published:** 1854-04

**Authors:** Wm. T. Russel

**Affiliations:** Canandaigua, New York.


### ARTICLE VII.

*An Essay on the Structure and Development of the Dental Tissues: Presented to the Faculty of the* BALTIMORE COLLEGE OF DENTAL SURGERY, FOR THE DEGREE OF DOCTOR OF DENTAL SURGERY, *March 1st*, 1854. By WM. T. RUSSEL, M. D., Canandaigua, New York.

WE propose, in this essay, to treat of the minute structure and development of the dental tissues—a subject which, even to this time, is involved in much obscurity. Our work, however, has been to set forth the main facts, free and isolated from their collaterals, which modern investigations have brought to knowledge, rather than to discuss the various hypotheses and theories that have been originated for their explanation.

Anatomy is, at all times, a demonstrative science, the great truths of which depend for their elucidation upon the revelations of the scalpel and microscope. Abstract and speculative reasonings can be of no avail, other than to assist in the interpretation of what may come within the field of vision; for, as it seems to us, much of *the mysterious* that prevailed in the sciences in former times, and to a great extent in our day, arises from the prurient desire on the part of some observers, to theorise instead of abiding time, and permitting "facts to speak for themselves," as made known.

Aiming to set forth ultimate truths, we have been obliged, necessarily, to confront a vast mass of diverse, and too often opposite opinions and observations; and here, always concisely as possible, we have stated each author's conclusions, without comment, to be confirmed or disproved by future investigations.

Nor is it without some degree of diffidence that we present this undertaking, fearing lest we may not have given that important consideration to certain views which they demand, or that our descriptions have not been sufficiently marked to portray the exact lineaments of the subject: but in reference to the one, we may recall to the minds of the learned, that much diversity of opinion among the great pioneers of dental microscopy exists in regard to the dental tissues; and as to the other, it is evidently impracticable to introduce in this place such plates and figures as may be necessary to illustrate the descriptions. It will be perceived, however, that we have made proper references, and in all cases "rendered honor to whom honor is due."

*The Structure and Development of Dental Tissues.*—The tissues that compose a tooth, are: the dentine, the enamel, the crusta-petrosa, or cementum, and the pulp.

*Dentine* was at one time supposed to be bone; but modern investigations go to show that it differs from that substance in its mode of formation, structure, and chemical composition, and, therefore, is entitled to a distinct rank among the other tissues of the body. It is the most internal of the hard structures of a human tooth, and composes the chief bulk of its body and root, and also gives to the organ its particular shape and density. In its centre is the pulp cavity, which, extending through the root and most of the body, opens externally at the apex of the former by a single aperture.

The *enamel* placed upon, and external to the dentine, covers the whole crown of a tooth after the manner of a cap. It is thickest upon the cusp, and gradually becomes thin on the sides, and toward the neck. This substance is by far the hardest of organized structures, and to such a degree is it possessed of this property, by which alone it is so beautifully adapted to its ultimate purposes of comminution and attrition, that finely tempered steel instruments produce but little impression upon it.

The *crusta-petrosa,* or cementum, is a thin lamina of true bone, which, together with the enamel, forms an encasement around the dentine. It surrounds the root, and extends a short distance within the pulp cavity. It is thinnest at the neck, and found in greatest abundance just below the middle of the root.

The *pulp* is, for the most part, composed of a very delicate reticulation of blood vessels and nerve filaments. It is situated in the pulp cavity, before noticed, and is essentially a centre of nutrition and sensation.

We have now in this general way given a hasty glance at the respective relations of the dental tissues, and as we progress, it will be found that in their formative and developmental processes, they are intimately associated with the epithelium, basement, and alveolar tissue of the great mucous system.

About the sixth week of fœtal life, according to Mr. Goodsir, is observed a slight "groove" or depression on the surface of the mucous membrane covering the rudimentary maxilla. It has a direction corresponding to the bone, and is bound in front and behind by a fold of the membrane. During the seventh week, a point merely may be seen, arising as it were from the bottom of the groove, which constitutes *the dental papilla.* Subsequently to this time, processes are sent off from each wall of the depression, which unite and thus surround the papilla, or as expressed by Mr. Nasmyth, form a "fence about it."

During the papillary period may be observed the incipient formation of the enamel pulp, as a "soft gelatinous substance," (Goodsir,) of whitish color, which encircles the base of the papilla like a belt, (Nasmyth.) The original point grows rapidly, its apex rising above the level of the follicle, and then, as by an arrest in the former, the walls of the latter enclose it, leaving, however, for a time, an aperture through which, as stated by Mr. Goodsir, a bristle may be passed, but which afterwards is closed by a coalition of the sides of the follicle. Together with the development of the papilla, proceeds also that of the enamel organ, so that when the former is surrounded by its sack, the space otherwise intervening between the two is filled up by the latter. We shall pass over the consideration of the organ just mentioned for the present, since at another time and place it will be duly noticed at some length.

The *structure* of the papilla during the follicular stage, is in a marked degree homologous to a tactile papilla, or a hair pulp; consequently, we observe in each an epithelial layer, a basement membrane, beneath which are found nucleated particles, and granular matter floating in a plastic lymph, and the submucous or alveolar tissue supporting the capillaries and nerves. It is, however, only during the early stage that the vessels and nerves are found at the base of the organ, for, as we shall see presently, they increase and occupy the body of the pulp as it is developed, and are distributed after a well determined and beautiful manner.

The changes which have taken place to form this new organ, like many other natural operations, are truly remarkable. The papilla, now enclosed in a sack, becomes the dentinal pulp. The mucous membrane is no longer recognized as such, its epithelium having disappeared or assumed another form, and consequently a new function; the basement, also, according to some authors, is absorbed, and its place in the reflected portion, supplied by another simple homogeneous membrane, whilst the submucous tissue now constitutes, for the most part, the dental capsule.

We will now examine this subject more minutely in the sacular or second stage. Upon a general view of the dentinal pulp, it is found to consist of blood vessels, nerves, alveolar tissue, plastic lymph, granular matter, nucleated cells, and an external membrane.

As before remarked, the capillaries occupy more of the body of the organ in this than in the follicular stage. They are distributed in a delicate alveolar tissue, and furnish the plastic material for the developmental cell process. (Todd & Bownan's Phys. Anat.) After dividing and subdividing extensively, they terminate in diminutive loops on the pulp surface.—(Nasmyth's Researches on the Teeth, pls. viii., ix.) Beyond these vascular limits, however, it is urged by some that there is another circulatory apparatus, in which normally is moving a white blood or serum, as in the cornea of the eye, which, being connected with the red capillaries, extend through the dentine, and is there distributed. That red blood may be observed sometimes in specimens of dentine when examined by the microscope, is a fact which in this day cannot for a moment be called in question. (See Harris' Prin. and Prac. of Dent. Surg.) But it does not seem to us a proper interpretation of it to infer that dentine, in a healthy condition, is supplied with red blood, for, so far as we have been able to inform ourselves, the specimens so examined were taken from teeth in a diseased state. Now, regarding the fact in the light of a natural dissection, we may suppose that by the morbid action going on in the organ, the circulators of white blood have been permitted, so to speak, by a law of the economy, when so circumstanced, to receive the red particles; or more plausibly still, by the same inflammatory process of the pulp, the red corpuscles are disintegrated and broken down, whilst the coloring matter is taken up and diffused, less or more, throughout the dentine, either with the agency of the dentinal tubuli or those diminutive channels just mentioned, which only exist as yet in hypothesis. All this, we are aware, in the present state of knowledge, is mere speculation, but it is the most satisfactory or specious explanation of the observations before noticed, and also of the peculiar pink hue which diseased teeth are sometimes known to assume.

The *nerves* of the pulp are distributed like the capillaries, and are said to terminate by fine filamentous loops, in the convexity of which "the white substance of Schwan" is not always to be detected. (Todd & Bowman's Phys. Anat.) That this ending is the ultimate limit of the nerve, is a point which we very much question. We now know that in different parts of the body, they terminate by single filaments, as in "the Pacinian corpuscles," and for anything known to the contrary, they may terminate in some such manner within, or upon the dentine. Though no one has ever demonstrated their presence to sight, it is very probable, nevertheless, that they do exist there. Every one is familiar with the fact that dentine is quite sensitive to the touch, even in a healthy condition, and becomes more so when subject to diseased action. Now, this is a physiological fact that can only be explained satisfactorily on the hypothesis, that the dentine is supplied in some manner with nerves, for as yet we have no knowledge that a function can be manifested without its proper organ to produce it.

The *plasma* is most probably the serum of the blood, furnished by the capillaries, and holds in solution the nutrient elements for the formation of the cells, as well as the earthy materials for the construction of dentine. The cells themselves originate, perhaps, directly from the proximate elements, and increase in number by the process of "division;" for if a pulp be examined from within toward the surface, its cells will be perceived to have a graduation of development, so to speak, from the simple aggregation of granular matter, up to the full sized cell.

The development of the pulp cells, Mr. Tomes divides into three stages; the first or alveolar stage consists of a "meshwork of delicate fibres and bands of homogeneous matter, in the thicker part of the walls of which are scattered here and there nucleated cells, while the meshes are occupied by a thick, clear homogeneous fluid or plasma, and in this are a number of nucleated cells." This stage precedes, and is preparatory of the next or cellular, of which he says, "the cells have no definite arrangement as regards each other, but are thickly scattered at pretty regular intervals throughout the section." In the third stage, the cells are arranged in a "linear succession," and have a direction vertical to the plane of the dentinal surface. (Tomes' Phys. and Surg. of Teeth, p. 85, 86.)

The views of Mr. Nasmyth are somewhat at variance, however, with those just noticed. According to this gentleman's observations, it appears that the cells, numerous and of infinite diversity of shape, "are evidently disposed in different layers throughout the body of the pulp," the deeper seated being "ill defined layers," whereas, those near the surface are arranged concentrically, and present in a section the form of "*reticular leaflets.*" (Nasmyth's Research on the Teeth, p. 139, fig. 29.) Whatever be the diversity of expressions on this subject, those cells which approach the pulp surface are described by all authors as having an arrangement in a linear succession, thus apparently seeking an adaptive purpose in reference to the formation of the dentine.

On the surface of the pulp is a transparent and homogeneous membrane, before alluded to, which, at a late period, is structureless, but when first observed, it is found to contain cells. (Nasmyth.) Upon its outside may be seen little pits or depressions, of an hexagonal shape corresponding to the endings of the tubuli; into those the enamel rods are inserted.

*Dentine.*—We observed in our first notice of the dental tissue, that this substance is regarded by some as only a modification of bone; and, indeed, if we adopt the views of those that support the tubular theory, there will be found a certain degree of analogy; thus, in the dentine we have a vascular membrane—the pulp, tubes or canals, and in some parts lacunæ, each of which have a counterpart in the Haversian system of true bone. But it is a little premature, perhaps, to pronounce positively upon this subject, since the very points of analogy are still under consideration by the investigators of dental tissues.

The *structure* of dentine, though investigated by Tomes, Owen, Todd, Bowman, Nasmyth, Arnold, Goodsir, and other eminent men, is still among "the vexed questions" of microscopy. It would be an undertaking far too extensive for our present purposes to pass in review the different opinions of all these gentlemen, and, therefore, confining our remarks to narrower limits, will describe without detail, the main features of the theories supported on the one hand by Mr. Tomes and others, and on the other by Mr. Nasmyth.

Dentine is composed of a tubular and an intertubular substance. The tubuli communicates with the pulp cavity, and radiate from it as a centre toward the dentinal surface. Their direction, however, are not in straight lines, but as remarked by Todd and Bowman, are curved like the italic letter *f*, and which also in turn are made up of smaller or secondary curves. From the main tubes pass off lateral branches, which, anastamosing with those of other tubuli, facilitate the intercommunication of dentine, and at the same time, by forming so many arches, contribute in a great degree to increase the strength and density of the tissue. The form and direction of the tubuli are not constant, but vary in different teeth, and in different parts of the same teeth; thus, a section from the cutting surface down to the pulp cavity, in a vertical direction, present the fibres in almost right lines, whereas on either side of the dental axis they are less or more curved, and between the roots of a molaris, they have been described by Mr. Tomes as possessing a spiral character. In some of the tubuli their branches go off in pairs, and are opposite; in others alternately one above the other from each side. There is an arrangement in the dentine corresponding in position to a fibre, which is not unlike a string of beads, each of which is represented by an original nucleus; such an arrangement, Mr. Tomes regards imperfect, the developmental process having been arrested. (Tomes' Dent. Phys. and Surg., p. 38, fig. 21.) For a more detailed account of the multitudinous forms of the dentinal fibres or tubuli, we must refer to the writings of Owen, Tomes and others.

The formation of dentine is another of those interesting natural operations, of which, as yet we have only outline notions. The deposit of the inorganic material is first observed in the transparent and amorphous membrane, at a point corresponding to the tip or cusp of the future tooth, and then progresses over the surface "down the sides and towards the centre" of the pulp. The cells in linear succession are placed against the inner surface of the dentinal membrane; their nuclei elongate, and at the point of contact the walls of each are absorbed, and thus form a continuous tube. The earthy matter is now deposited within the cell membrane—around the tube—and in the densest and most compact part of the fully formed dentine; this is "the tubular substance."

The intertubular tissue is formed by the deposit of inorganic matter in the plasma, between the cells, after the manner somewhat of bone formation, and like the latter, in its ultimate texture is supposed to be granular. In this case the plasma is to the dentine what cartilage is to bone—an hypothesis which waits further investigation.

We have all along assumed that the dentine is furnished with tubes having a definite calibre; and the ground for believing that they are such, we may sum up in a quotation from a learned source: "On the surface of the pulp cavity, the orifices of the tubuli can be seen; and in transverse sections of the tubuli, their proper walls, the width of their walls and calibre and their distance apart are all discoverable." "Their hollowness is proved by the chasing of bubbles along them, visible under the microscope when turpentine is added to a dry section, and also, by the gas which may be seen to be disengaged in bubbles, chiefly from their interior, when a section is similarly treated with acid." "We do not regard the tubuli as filled up by solid contents, but as possessing a truly hollow bone designed to give passage to fluid." (See Todd & Bowman's Phys. Anat. p. 530, also Tome's Dent. Phys. and Surg., pp. 39, 41, 42.)

These evidences are stated as facts made known by the microscope, and their truth or error rests solely upon future investigation. Mr. Nasmyth objects to the tubular theory of dentine, if we may so call it, and urges that the appearance of tubes is wholly deceptive, arising from the manner of preparing the specimen for observation; and, consequently, has conceived another and better method than that usually followed, by which he is enabled to present the true and constant structure of this tissue. (Research on the Teeth.)

The dentine being formed from the pulp by a deposition of calcareous matter in its cells, retains their persistent reticular arrangement in concentric layers. In corroboration of this assertion, a transverse section of dentine in very thin lamina, when viewed under the microscope, presents an infinitude of little "circles which intersect one another in all directions;" and a vertical section viewed in like manner, presents the faint outline of the cell parietes, surrounding the earthy matter; and both of these arrangements correspond to that of the original pulp cells as supported by our author. Now, if the last section be submitted to acid, so as to remove the earthy constituents, and attentively observed, there will pass in the field of vision a succession of disclosive scenes, which are demonstrative of the truth of our previous remarks. At first, the acid removing a part of the earthy salts, leaves the cell walls surrounding the inorganic substance, and having an arrangement of the "reticular leaflets;" next, when nearly all the earths are removed, the fibres are seen as lines, from the sides of which extends little processes, supposed to be the remnant of the cell walls; and lastly, when all the earths and cell walls have been dissolved and washed away, there is left the isolated fibre made up of the persistent solid nuclei, arranged in "the linear succession," and hence called by Mr. Nasmyth "the baccated fibre."

Now it would appear from the above statements, that the cells and nuclei retain their integrity as such throughout, and at the proper period of the developmental process the former receive earthy matter, and are most probably the true formative organs of dentine; whilst the latter, still occupy their position in the sides of the cells, and constitute the baccated fibre for the transmission, it may be, of a nutritive fluid.

It is contended by our author, that the tubuli, so called, are not in truth tubes, but are fibres, such as we have just described. Among other reasons for this belief we may briefly state the following: 1st, that fluids can *not be made to enter them;* 2dly, *compression* of them does not manifest the same appearances, which well defined tubes do when similarly treated. 3dly, The diameters of the fibres correspond to the admeasurements assigned by others to the tubuli, and 4thly, transverse sections when viewed under the microscope do not present any evidence of apertures or orifices of true tubes. (Nasmyth's Researches on the Teeth.)

We have now hastily glanced at the two theories of the formation and structure of the dentine; and as we observed in our first remarks, opinions on subjects of this character can only be verified by demonstration from future investigations; we shall, therefore, pass on to the consideration of the next tissue in order.

*Enamel.*—This substance, placed upon and external to the dentine, possesses a smooth and polished surface, is of a flinty hardness, capable of resisting the edges of the best tempered steel. It varies in color in different individuals, from a chalky whiteness to a dark yellow, all of which shades are marked and characteristic indications of the kind of teeth, as well as the amount of constitutional vigor at the time of their formation. Enamel is the thickest on the cusp, and becomes thin in the depressions of the grinding surface, and on the sides toward the neck of the tooth, where it exists only as an attenuated lamella. In transverse sections, it presents an arrangement into hexagonal compartments, each of which is the end of an enamel rod. Viewed in a vertical section, the rods seem to be placed between two lines, and are marked into divisions; but Mr. Nasmyth describes them as made up of cells of a semi-circular shape, the convexities of which look toward the external surface. They are for the most part parallel with each other, and rest in the depressions on the dentinal surface, which we have already described. In the newly formed tissue, distinct tubes may be seen in it, which at a late period are obliterated or filled. (Tomes.)

Whilst treating of the papillary stage, we referred to a little ring of whitish matter, about the base of the papilla, the early appearance of which was first noticed by Mr. Nasmyth, but at a later period of development, was previously described by Mr. Goodsir as "the gelatinous granular substance;" this is the enamel organ. When the papilla is enclosed in its sac, this organ occupies the space between the two, and rests closely upon the former as a cap or "hood." (Raschkow.)

The structure of the enamel pulp, like that of the dentine is cellular, and its development, for descriptive purposes, is said to take place by three stages, which we will, without detail, now notice. And first: the granules and peculiar nucleated cells float in their plasma, in the reticulations of a "very fine structureless fibre, which proceeds from the membrane of the sac," and through which also ramify "minute blood vessels." (Tomes.) Secondly, by a gradual transformation apparently, the first stage passes into the second, in which the fibres instead of having an irregular arrangement, "radiate from central nuclei, take a straight course, and join other nuclei that are similarly circumstanced." In consequence of this radiate appearance, their fibres are called *the stellate tissue;* and along with its cells and nuclei constitutes the enamel *pulp* of Todd and Bowman, in which also Mr. Tomes includes the reticular tissue of the first stage. The cells are of an oval shape, and contain nuclei, and nucleoli; they increase in number by an accession of cells resembling their nuclei. Thirdly, this stage is formed from the last, (Todd and Bowman,) and is made up of a layer of nucleated cells of a flattened elongated shape, upon which rests a columnar epithelium.

These form the enamel matrix or membrane of some authors, and correspond to "the villous surface" described by Mr. Goodsir. (Tomes' Dent. Phys. & Surg., p. 97, fig. 54.)

Mr. Nasmyth describes the enamel pulp as composed of three kinds of cells; the first class found in the interior, are flat and triangular in form, and are connected to adjacent cells by means of delicate filaments prolonged from one of their angles. The second class are situated on the superficial and deep aspect of the latter, are of an oval shape and form an envelop to the preceding. The third class correspond to the epithelial layer before mentioned, with the exception that the nuclei of the cells are not persistent. (Nasmyth's Researches on the Teeth, p. 105.)

We do not feel ourselves able to harmonize the opinions of Mr. Tomes and Mr. Nasmyth, unless the first class of cells of the latter gentleman correspond with the transition of the first stage into the second of the former; for as it seems to us, Mr. Tomes includes in his description of the structure of the enamel pulp, the reticular tissue in which "minute vessels ramify," which Mr. Nasmyth regards as belonging to the dental sac, or as we should say, corresponds to the structure on the inner surface of the sac, as made out by the last gentleman. If this remark be correct, then the one opinion corroborates the other of the structure of the pulp.

Calcification, or the formation of enamel, begins first on the dentinal surface, by a deposit of inorganic matter in the columnar cells, and progresses by the superposition of other and similar cells, till the process is completed. The origin of the earthy matter is evidently from the blood; but do the vessels transmit the earthy salts in solution by the agency of cells to the desired point, or do they ramify on the enamel membrane? The last view, first published by Raschkow, we believe, has been corroborated in a series of experiments upon the young of the inferior animals, as well as in the human fetus by Professor Harris; and at this time is strenuously supported in his lectures. (See also Harris' Princ. & Prac. of Dent. Surg.) The opinion expressed in the first query is also supported by Tomes, Nasmyth, Owen and others.

May not this diversity of opinion arise from the different periods of the developmental process at which the examination is made? At an early period of the enamel pulp, as we have seen, it is composed of a plasma for the most part, and cells which are in process of development for the formation of the enamel; at the same time, it takes place at the expense of the plasma, or in other words, the plasma is consumed, as may be proved by observation; and in consequence of these changes, the vascular layer of the sac approaches the enamel membrane, and eventually is brought into immediate contact with it, so that in consequence of this relation, the enamel membrane would put on the appearance of being traversed with vessels.

Crusta-petrosa, or the cementum, is essentially an osseous tissue, possessing, however, less of its marked characters in some parts depending on its extreme thickness. It is situated in the alveolus, surrounding closely the root of the tooth, and extends a short distance into the orifice of the pulp cavity. The ultimate structure of cementum is granular, and when found in abundance is laminated, and traced by vasculur canals from which radiate tubuli as in bone. Like this substance, also, cementum contains lacunæ whose canaliculi on the outer surface are directed toward the alveolar walls: but within they are, less or more, connected with the granular layer of dentine, as well as the dentinal tubuli. (Tomes.) But this idea would lead to the notion of a vital connection between the cement and dentine; a relation which Mr. Nasmyth denies, and affirms, "that its union is one of mechanical contact, and its purpose purely mechanical." (Nasmyth's Researches on Teeth, p. 81.) The formation of the cementum, like the other dental tissues, is by cell agency or a pulp. It is placed in the alveolus between its periosteum and the dentinal pulp, and composed of an areolar tissue connected with the former, the interstices of which contain the plasma, granules and nucleated cells.

The nucleated particles are of an oval shape, and arranged at right angles to the dental axis. Ossification first occurs in the granular matter about the neck of the tooth, where few cells only are found, and then progresses on the dentinal surface of the pulp, involving the cell walls, whilst the nuclei retain their persistence, and eventually become the lacuna, before mentioned. The development of the cement is from within outward, and its plasma sustains a relation to it that the temporary cartilage do to the flat bones, for upon each, whilst the convertible material is forming externally, it is being transformed within, into the more permanent tissue.

In conclusion, we have now attempted, concisely as is practical, to present a statement of some of the more modern investigations of the structure and development of the dental tissues; and imperfectly accomplished as our part may be, it is hoped that we have given a correct interpretation of each opinion, and in no instance, sacrificed or distorted the truth.

